# Supplementary material for: Psychological Distress Related to the COVID-19 Pandemic: The Protective Role of Hope
Source: Eur J Investig Health Psychol Educ. 2023 Jan 3;13(1):67–80. doi: 10.3390/ejihpe13010005 (PMC9857999; doi:10.3390/ejihpe13010005)
Supplement: Supplementary file 1 [file ejihpe-13-00005-s001.zip › ejihpe-2073142-supplementary.pdf]

Supplementary Materials

Table S1. Distribution of Demographics according to Hope and Psychological Distress

Demographics		Agency				Pathways				Hope Overall				Psychological Distress			
		M	SD	t/F	p	M	SD	t/F	p	M	SD	t/F	p	M	SD	t/F	p
Gender	Male	20.13	3.97	2.24	.03	20.06	3.76	2.37	.02	43.75	8.28	2.33	.02	27.26	16.12	-2.22	.03
	Female	19.34	3.82			19.18	4.35			41.71	10.58			30.56	16.66		
Age	Young Adults (18-35)	19.84	3.76	2.25	.03	19.82	3.71	2.42	.02	43.19	8.78	3.55	.00	28.36	19.33	-4.75	.00
	Middle Age Adults (36-55	18.80	4.44			18.64	5.48			39.13	12.90			33.60	18.63		
Education	Intermediate	18.90	3.88	3.60	.03	18.92	4.85	5.34	.01	36.30	11.65	20.09	.00	33.96	17.92	12.02	.00
	Graduation	19.42	4.12			19.16	3.96			42.84	8.82			29.02	18.02		
	Master	20.15	3.64			20.27	3.82			44.24	8.97			27.90	10.54		
Residence	Urban	19.92	6.42	-3.18	.00	19.85	6.10	-2.76	.01	39.76	11.57	-3.27	.00	33.22	17.31	6.48	.00
	Rural	21.61	5.22			21.22	4.73			42.83	8.77			23.98	13.77		
Family System	Nuclear	20.20	3.76	2.20	.03	20.33	4.13	3.10	.00	44.67	8.76	3.64	.00	27.20	17.32	-3.53	.00
	Joint	19.39	3.94			19.13	4.09			41.40	9.97			30.29	18.08		
Marital Status	Single	20.63	3.46	8.58	.00	19.69	3.99	4.77	.01	43.39	9.95	7.79	.00	28.31	19.75	8.72	.00
	Married	16.63	3.28			19.34	4.17			39.81	7.70			32.27	17.47		
	Divorced	14.00	5.19			15.70	6.93			36.50	11.50			33.77	16.06		
